# Cardioprotective effects of Cu^(II)^ATSM in human vascular smooth muscle cells and cardiomyocytes mediated by Nrf2 and DJ-1

**DOI:** 10.1038/s41598-016-0012-5

**Published:** 2016-12-21

**Authors:** Salil Srivastava, Philip J. Blower, Aisah A. Aubdool, Robert C. Hider, Giovanni E. Mann, Richard C. Siow

**Affiliations:** 1 Cardiovascular Division, British Heart Foundation Centre of Research Excellence, Faculty of Life Sciences & Medicine, King’s College London, 150 Stamford Street, London, SE1 9NH UK; 2 Imaging Sciences & Biomedical Engineering Division, British Heart Foundation Centre of Research Excellence, Faculty of Life Sciences & Medicine, King’s College London, The Rayne Institute, St. Thomas’ Hospital, London, SE1 7EH UK; 3 Institute of Pharmaceutical Science, Faculty of Life Sciences & Medicine, King’s College London, 150 Stamford Street, London, SE1 9NH UK

## Abstract

Cu^(II)^ATSM was developed as a hypoxia sensitive positron emission tomography agent. Recent reports have highlighted the neuroprotective properties of Cu^(II)^ATSM, yet there are no reports that it confers cardioprotection. We demonstrate that Cu^(II)^ATSM activates the redox-sensitive transcription factor Nrf2 in human coronary artery smooth muscle cells (HCASMC) and cardiac myocytes (HCM), leading to upregulation of antioxidant defense enzymes. Oral delivery of Cu^(II)^ATSM in mice induced expression of the Nrf2-regulated enzymes in the heart and aorta. In HCASMC, Cu^(II)^ATSM increased expression of the Nrf2 stabilizer DJ-1, and knockdown of Nrf2 or DJ-1 attenuated Cu^(II)^ATSM-mediated heme oxygenase-1 and NADPH quinone oxidoreductase-1 induction. Pre-treatment of HCASMC with Cu^(II)^ATSM protected against the pro-oxidant effects of angiotensin II (Ang II) by attenuating superoxide generation, apoptosis, proliferation and increases in intracellular calcium. Notably, Cu^(II)^ATSM-mediated protection against Ang II-induced HCASMC apoptosis was diminished by Nrf2 knockdown. Acute treatment with Cu^(II)^ATSM enhanced the association of DJ-1 with superoxide dismutase-1 (SOD1), paralleled by significant increases in intracellular Cu^(II)^ levels and SOD1 activity. We describe a novel mechanism by which Cu^(II)^ATSM induces Nrf2-regulated antioxidant enzymes and protects against Ang II-mediated HCASMC dysfunction via activation of the Nrf2/DJ-1 axis. Cu^(II)^ATSM may provide a therapeutic strategy for cardioprotection via upregulation of antioxidant defenses.

## Introduction

Reactive oxygen species (ROS) are important mediators of signaling in the cardiovascular system which are generated by endothelial and smooth muscle cells (SMC) and cardiomyocytes. Excessive ROS generation results in oxidative stress that drives the progression of pathophysiological events integral to the development of cardiovascular diseases such as hypertension, atherosclerosis, and cardiomyopathy. Angiotensin II (Ang II), the active component of the renin angiotensin system, increases ROS generation, resulting in SMC dysfunction contributing to cardiovascular disease^1–3^.

In response to oxidative stress, the redox sensitive transcription factor NF-E2 related factor 2 (Nrf2) orchestrates the expression of endogenous antioxidant defence enzymes^4^. Under homeostatic conditions, Nrf2 is repressed by Kelch-like ECH-associated protein-1 (Keap1) and targeted for ubiquitin mediated proteasomal degradation. The activation of Nrf2 occurs following the modification of reactive cysteines on Keap1, resulting in the nuclear accumulation of Nrf2^5^, binding to the antioxidant response element (ARE) in the promoter region of target antioxidant defense genes such as heme oxygenase-1 (HO-1), NADPH quinone oxidoreductase-1 (NQO1), peroxiredoxin 1 (Prx1), and the glutamate cysteine ligase modifier subunit (GCLM), an essential enzyme for glutathione (GSH) synthesis^6–8^. Nrf2 has become a focus for therapeutic interventions due to its activation by a range of pharmacological agents and natural compounds in addition to oxidative stress^9^. However, Nrf2 activation is dependent upon its cytoplasmic stabilisation by the multifunctional Parkinson’s-associated protein DJ-1^10^, which also acts as a copper chaperone, enhancing cytosolic superoxide dismutase-1 (SOD1) function^11,12^.

Recently, the copper^II^-bisthiosemicarbozonato complex Copper(II)-diacetyl-bis(N4-methylthiosemi-carbazone) [Cu^(II)^ATSM] (Fig. S1A), a hypoxia sensitive positron emission tomography imaging agent^13^, has been reported to protect against oxidative damage arising from Parkinson’s disease (PD)^14^ and amyotrophic lateral sclerosis (ALS) in a therapeutic regime *in vivo* ^13,15^. However, the mechanisms by which Cu^(II)^ATSM confers protection against oxidative injury remain to be fully elucidated. To date, there are no reports on the potential of Cu^(II)^ATSM to enhance the expression and activity of endogenous antioxidant defense enzymes regulated by Nrf2/DJ-1 signalling in the cardiovascular system. We have investigated for the first time whether treatment of human coronary artery SMC (HCASMC) and cardiomyocytes (HCM) with Cu^(II)^ATSM induces expression of antioxidant enzymes via activation of Nrf2 and its co-activator protein DJ-1, thereby providing protection against the pro-oxidant effects of Ang II, including SMC apoptosis, proliferation and increased intracellular calcium^16–19^. Notably, we show that oral administration of Cu^(II)^ATSM in mice induces antioxidant defense enzymes in the heart and aorta *in vivo*, and treatment of HCASMC and HCM *in vitro* with Cu^(II)^ATSM activates the Nrf2-DJ-1 axis to upregulate antioxidant protein expression. We further report that pre-treatment of HCASMC with Cu^(II)^ATSM affords protection against the pro-oxidant actions of Ang II^1,2,20^. By enhancing the association of DJ-1 with SOD1 and increasing SOD1 activity, Cu^(II)^ATSM may confer cardiovascular protection through activation of antioxidant defenses mediated by the Nrf2/DJ-1 axis.

## Results

### Cu^(II)^ATSM induces expression of endogenous antioxidant proteins in HCASMC via Nrf2

**Figure 1:**
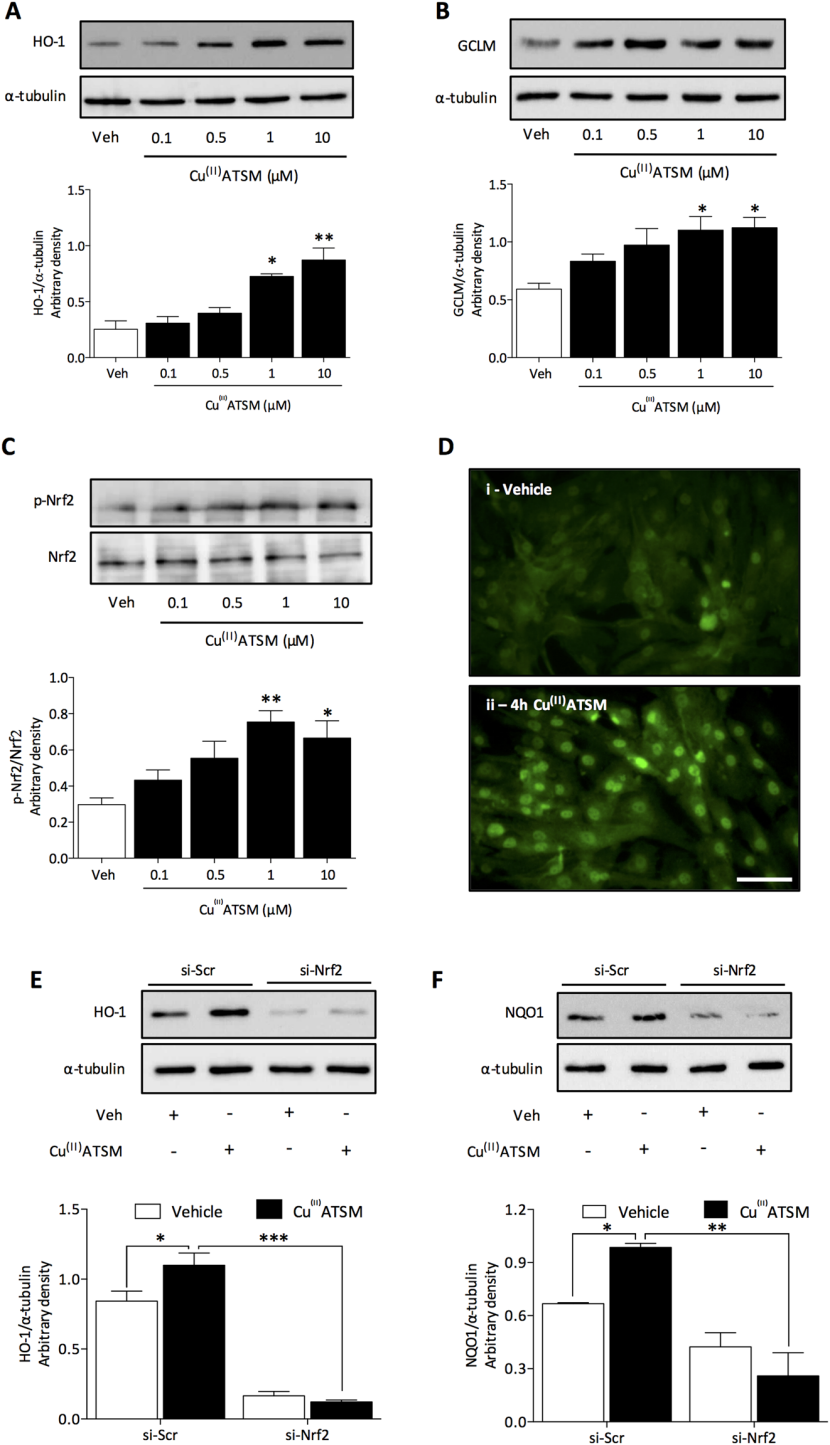
Cu^(II)^ ATSM induces nuclear translocation of Nrf2 and antioxidant protein expression in HCASMC. HCASMC were treated with Cu^(II)^ATSM (0.1, 0.5, 1 and 10 µM) for 12 h and expression of (**A**) HO-1 and (**B**) GCLM assessed by immunoblotting with densitometric analysis relative to α-tubulin. Data denote mean ± S.E.M., n = 5, *P < 0.05, **P < 0.001 vs vehicle (one-way ANOVA and Bonferroni *post hoc* analysis). (**C**) HCASMC were treated with Cu^(II)^ATSM (0.1, 0.5, 1 and 10 µM, 30 min) and phosphorylation of Nrf2 at serine 40 assessed by immunoblotting with densitometric analysis relative to total Nrf2. Data denote mean ± S.E.M., n = 4, *P < 0.05, **P < 0.001 vs vehicle (one-way ANOVA and Bonferroni *post hoc* analysis). (**D**) HCASMC were treated with Cu^(II)^ATSM (1 µM, 4 h) and Nrf2 localisation assessed by fluorescence microscopy (scale bar = 5 µm). Induction of (**E**) HO-1 and (**F**) NQO1 protein in response to Cu^(II)^ATSM (1 µM, 12 h) was assessed following transient transfection of cells with scramble (Scr) si-RNA or Nrf2 siRNA. Data denote mean ± S.E.M., n = 4, *P < 0.05, ***P < 0.001 (two-way ANOVA and Bonferroni *post hoc* analysis).

In order to assess concentration dependent induction of antioxidant defense enzymes by Cu^(II)^ATSM, HCASMC were treated with Cu^(II)^ATSM (0.1–10 µM, 12 h). A significant upregulation of HO-1 (Fig. 1A) and GCLM (Fig. 1B) protein expression was observed at concentrations of 1 µM and 10 µM. Treatment of cells for 12 h with equivalent concentrations of the bis(thiosemicarbazone) ligand ATSM alone had negligible effects on HO-1 or GCLM expression (Fig. S2), suggesting that Cu^(II)^ is required in the ATSM complex to mediate induction of these proteins. Levels of the intracellular antioxidant GSH^6^ were significantly (P < 0.05, n = 5) increased following Cu^(II)^ATSM (1 µM, 12 h) treatment (17.3 ± 1.71 nmol/mg protein) compared to vehicle (12.1 ± 0.8 nmol/mg protein). To determine whether the observed induction of antioxidant proteins by Cu^(II)^ATSM was mediated via Nrf2, we examined Ser40 phosphorylation of Nrf2^21^. Treatment of HCASMC with Cu^(II)^ATSM (0.1–10 µM, 30 min) resulted in a concentration dependent increase in Nrf2 phosphorylation (Fig. 1C). Moreover, treatment of HCASMC with Cu^(II)^ATSM (1 µM, 4 h) induced nuclear translocation of Nrf2 determined by immunofluorescence (Fig. 1D) and by immunoblotting of nuclear lysates (Fig. S3A). Notably, knockdown of Nrf2 (Fig. S3B) attenuated Cu^(II)^ATSM (1 µM, 12 h) mediated induction of HO-1 (Fig. 1E) and NQO1 (Fig. 1F) protein expression in HCASMC, demonstrating that Nrf2 activation underlies the induction of key antioxidant proteins by Cu^(II)^ATSM.

### Cu^(II)^ATSM does not affect ATP levels or cell viability

Excess intracellular Cu^(II)^ levels are known to cause mitochondrial toxicity and dysfunction^22^. As mitochondria are the major source of ATP^23^, cellular ATP content was assessed in HCAMSC. Treatment with Cu^(II)^ATSM (1 µM, 8 h) did not perturb ATP levels (P > 0.05, n = 4) in cells treated with Cu^(II)^ATSM (0.65 ± 0.07 μmol/mg protein) compared to vehicle treatment (0.54 ± 0.06 μmol/mg protein). Furthermore, using the MTT assay, it was evident that treatment of HCAMSC with Cu^(II)^ATSM (0.1–1 µM, 24 h) did not affect cell viability (Fig. S4).

### Cu^(II)^ATSM induces antioxidant protein expression in human cardiomyocytes and *in vivo*

**Figure 2:**
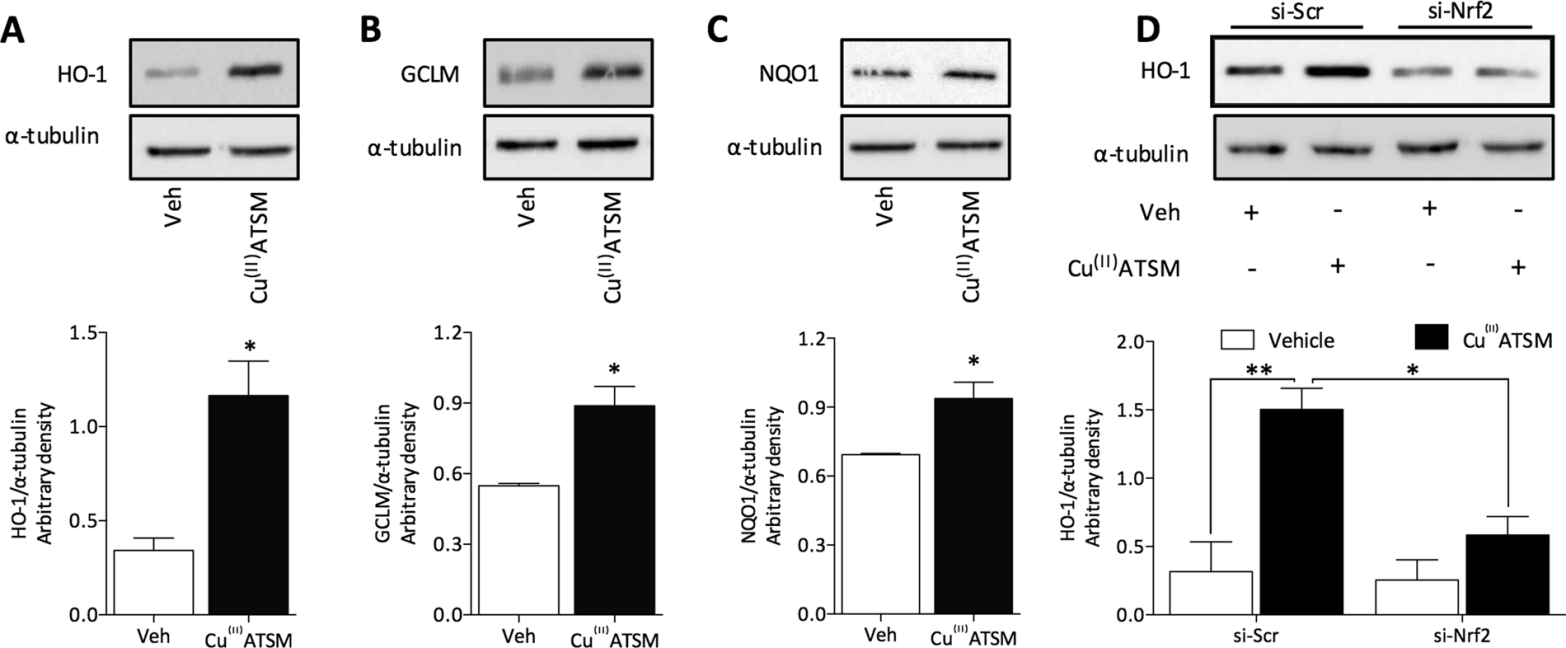
Cu^(II)^ATSM induces Nrf2 target antioxidant protein expression in HCM *in vitro*. HCM were treated with Cu^(II)^ATSM (1 µM, 12 h) and expression of (**A**) HO-1, (**B**) GCLM and (**C**) NQO1 assessed by immunoblotting with densitometric analysis relative to α-tubulin. Data denote mean ± S.E.M., n = 4, *P < 0.05 vs vehicle (Student’s t-test). **(D)** Induction of HO-1 in HCM in response to Cu^(II)^ATSM (1 µM, 12 h) following transient transfection of cells with scramble (Scr) si-RNA or Nrf2 si-RNA. Data denote mean ± S.E.M., n = 4, *P < 0.05, **P < 0.01 (two-way ANOVA and Bonferroni *post hoc* analysis).

**Figure 3:**
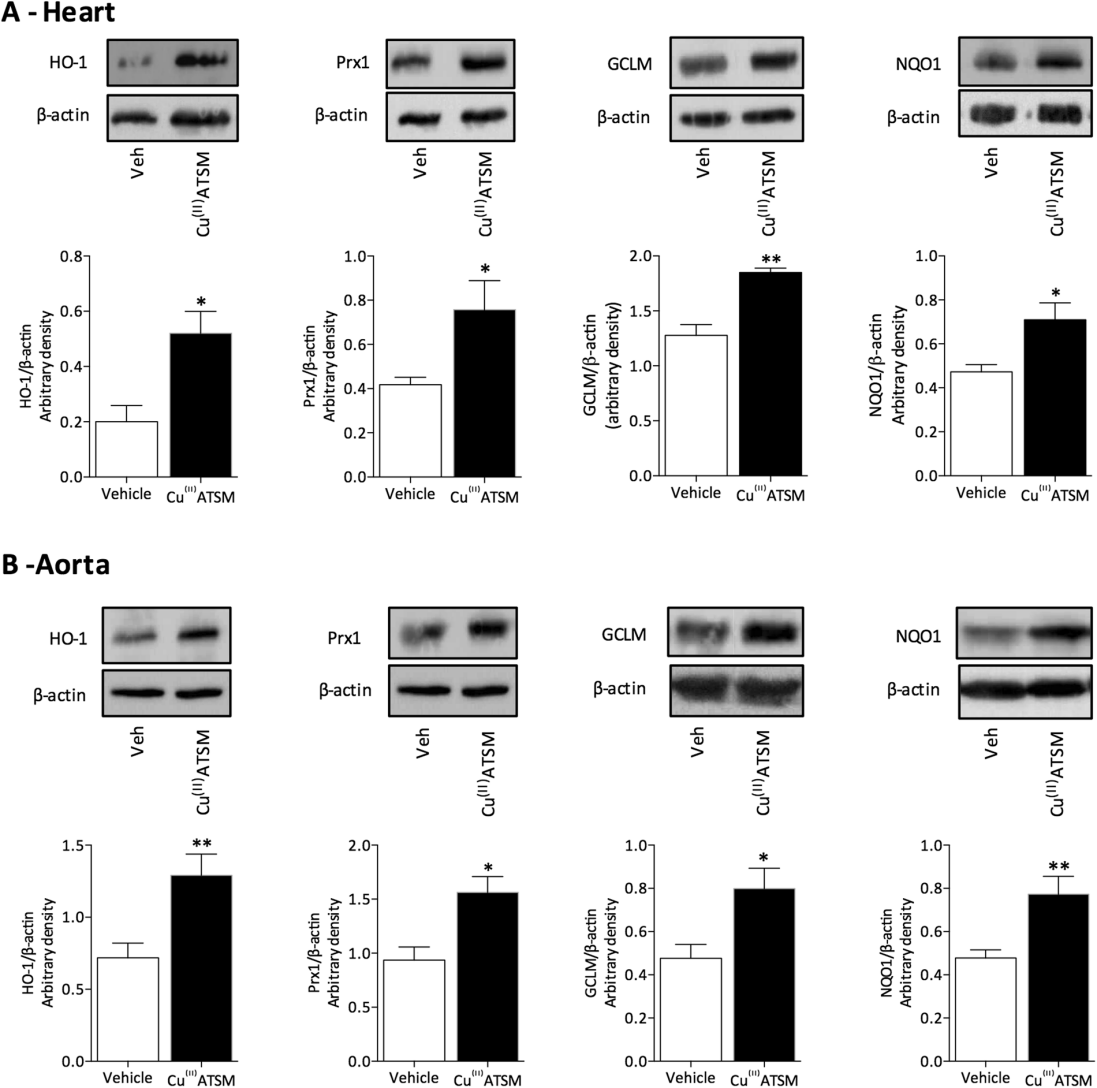
Oral delivery of Cu^(II)^ATSM induces Nrf2 target antioxidant protein expression in murine heart and aorta. Heart (**A**) and aortic (**B**) tissue homogenates from C57/BL6 male mice administered Cu^(II)^ATSM by oral gavage (30 mg/kg) were immunoblotted for HO-1, Prx1, GCLM and NQO1. Data denotes mean ± S.E.M., n = 5 animals per group, *P < 0.05 and **P < 0.01 vs vehicle (Student’s t-test).

In addition to the induction of antioxidant proteins in HCASMC, Cu^(II)^ATSM (1 μM, 12 h) significantly induced HO-1 (Fig. 2A), GCLM (Fig. 2B) and NQO1 (Fig. 2C) expression in HCM. Notably, knockdown of Nrf2 in HCM abolished Cu^(II)^ATSM mediated induction of HO-1. To further verify our *in vitro* data, we examined the effect of Cu^(II)^ATSM delivery by oral gavage in mice on antioxidant protein expression in the heart (Fig. 3A) and aorta (Fig. 3B). Cu^(II)^ATSM was delivered by oral gavage at a dose of 30 mg/kg, which has previously been reported to confer protection against oxidative stress *in vivo* ^13–15^. A significant increase in HO-1, Prx1, GCLM and NQO1 protein expression was observed in heart and aortic tissue at 24 h after oral administration of Cu^(II)^ATSM. These findings provide the first evidence that Cu^(II)^ATSM enhances Nrf2-regulated antioxidant protein expression in HCASMC and HCM *in vitro* and in the murine heart and aorta *in vivo.*

### DJ-1 is required for induction of HO-1 by Cu^(II)^ATSM

**Figure 4:**
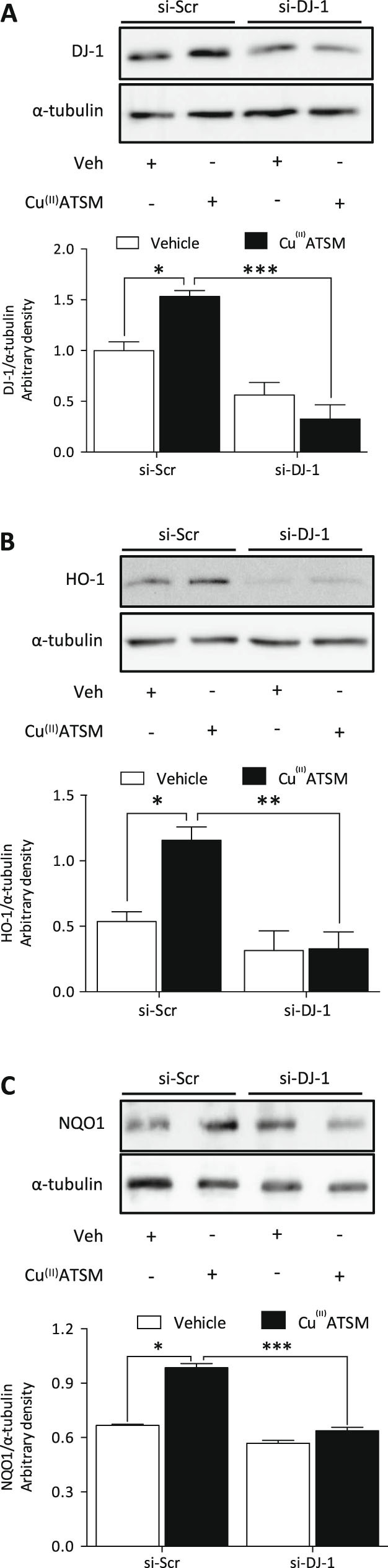
Cu^(II)^ATSM mediated induction of HO-1 expression in HCASMC is dependent on DJ-1. Induction of (**A**) DJ-1, (**B**) HO-1 and (**C**) NQO1 by Cu^(II)^ATSM (1 µM, 12 h) was assessed in HCASMC transiently transfected with scramble (Scr) si-RNA or DJ-1 si-RNA, and expression determined by immunoblotting relative to α-tubulin. Data denote mean ± S.E.M., n = 4, P < 0.05, **P < 0.01, ***P < 0.001 (two-way ANOVA and Bonferroni post hoc analysis).

Nrf2 stability and transcriptional activity are known to be enhanced by DJ-1^10^, and in the present study a significant increase in DJ-1 protein expression was observed in HCASMC following Cu^(II)^ATSM treatment (1 µM, 12 h) which was attenuated following DJ-1 knockdown (Fig. 4A). Silencing of DJ-1 attenuated basal, albeit not significantly, and Cu^(II)^ATSM (1 µM, 12 h) induced HO-1 (Fig. 4B) and NQO1 (Fig. 4C) protein expression. These findings provide the first evidence that DJ-1 plays a critical role in the induction of antioxidant proteins by Cu^(II)^ATSM, likely through its ability to stabilize and enhance Nrf2 activity^24,25^.

### Cu^(II)^ATSM reduces angiotensin II-mediated superoxide generation

**Figure 5:**
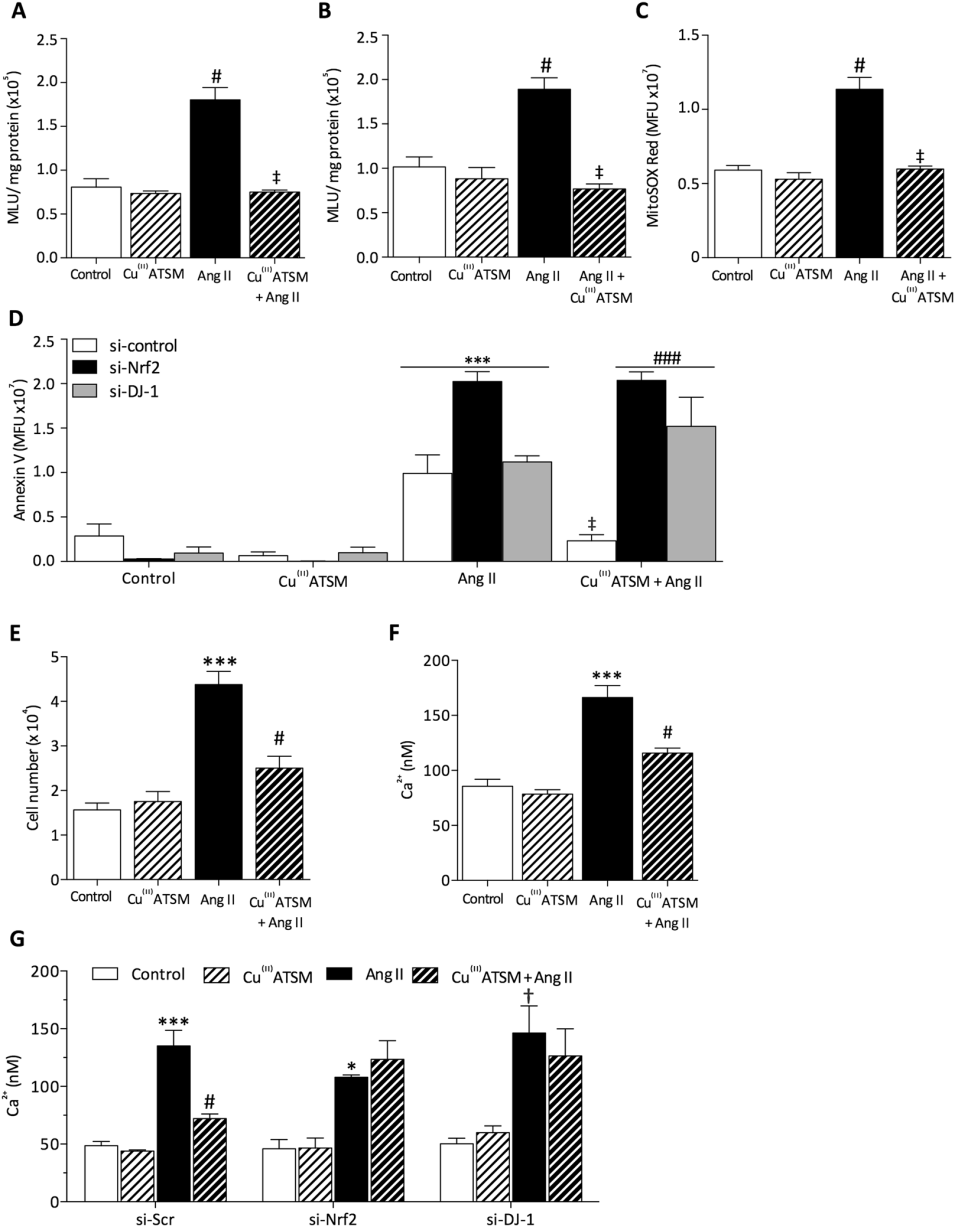
Cu^(II)^ATSM pre-treatment protects against Ang II-mediated superoxide generation, apoptosis, proliferation and intracellular Ca^2+^ elevation. (**A**) HCASMC were pre-treated with Cu^(II)^ATSM (1 µM, 12 h) and then with angiotensin II (Ang II, 200 nM, 4 h). Superoxide generation was assessed using L-012 enhanced chemiluminescence measured over 10 min. (**B**) HCASMC were treated with 200 nM Ang II for 4 h, prior to superoxide measurement in the presence of Cu^(II)^ATSM (1 µM). (**C**) Mitochondrial superoxide generation was determined using MitoSOX red in HCASMC following treatment with Ang II (200 nM, 4 h) and subsequent treatment with Cu^(II)^ATSM (1 µM, 30 min). Data denote mean ± S.E.M., n = 4–6, ^#^P < 0.01 vs control, ^‡^P < 0.01 compared to Cu^(II)^ATSM treatment, (one-way ANOVA and Bonferroni *post hoc* analysis), (**D**) Annexin V fluorescence was used to assess Ang II (200 nM, 12 h) induced apoptosis in HCASMC following Nrf2 or DJ-1 siRNA knockdown and pre-treatment with Cu^(II)^ATSM (1 µM, 12 h). Data denote mean ± S.E.M., n = 4, ***P < 0.001 vs control, ^‡^P < 0.001 vs cells treated with Ang II in the absence of Cu^(II)^ATSM, ^###^P < 0.001 vs cells treated with Cu^(II)^ATSM only (one-way ANOVA and Bonferroni post hoc analysis). (**E**) HCASMC proliferation was assessed following pre-treatment with Cu^(II)^ATSM (1 µM, 12 h) prior to Ang II (200 nM, 72 h). (**F**) Ang II (200 nM, 30 min) mediated changes in intracellular Ca^2+^ (Fura-2AM fluorescence) in HCASMC pretreated with Cu^(II)^ATSM (1 µM, 12 h). Data denote mean ± S.E.M., n = 4, ***P < 0.001 compared to control, ^#^P < 0.001 vs Ang II (one-way ANOVA and Bonferroni post hoc analysis).

As Ang II is known to induce vascular superoxide generation^2^, we assessed whether Cu^(II)^ATSM pre-treatment attenuated Ang II-mediated oxidative stress. HCASMC were treated with Ang II (200 nM, 4 h) and superoxide generation was detected in live cells by L-012 enhanced chemiluminescence. Pre-treatment of cells with Cu^(II)^ATSM (1 μM, 12 h) prior to Ang II exposure significantly reduced Ang II-induced superoxide generation (Fig. 5A). As treatment with Cu^(II)^ATSM alone did not alter the basal levels of superoxide, this suggests that upregulation of endogenous antioxidant defense enzymes by Cu^(II)^ATSM pre-treatment contributes to the attenuation of Ang II-induced oxidative stress. Furthermore, acute treatment with Cu^(II)^ATSM (1 µM, 30 min) also attenuated Ang II-induced superoxide generation (Fig. 5B).

Since Ang II is known to increase superoxide generation through mitochondrial activity^26^, we examined the effect of acute Cu^(II)^ATSM treatment on Ang II-induced mitochondrial superoxide generation using MitoSOX red fluorescence in HCASMC (Fig. 5C and Fig. S5). Ang II treatment (200 nM, 4 h) significantly increased mitochondrial superoxide generation, which was significantly reduced following acute Cu^(II)^ATSM (1 µM, 30 min) treatment. Treatment with Cu^(II)^ATSM alone did not alter basal levels of mitochondrial superoxide generation.

### Cu^(II)^ATSM protects against angiotensin II-induced apoptosis via Nrf2 and DJ-1

As Ang II has been reported to elicit apoptosis in VSMC^27^, we used annexin V binding as an index of apoptosis to examine whether Nrf2 or DJ-1 mediates protection afforded by Cu^(II)^ATSM against Ang II in HCASMC (Fig. 5D and Fig. S6). Pre-treatment with Cu^(II)^ATSM (1 µM, 12 h) prior to Ang II (200 nM, 12 h) significantly reduced levels of Ang II-induced apoptosis. Cu^(II)^ATSM treatment alone did not enhance apoptosis (Fig. 5D), further demonstrating that cell viability was unaltered. Cu^(II)^ATSM-mediated protection against Ang II-induced apoptosis was attenuated following Nrf2 or DJ-1 knockdown, establishing that both DJ-1 and Nrf2 are required for Cu^(II)^ATSM-mediated protection against Ang II-induced HCASMC apoptosis.

### Cu^(II)^ATSM reduces angiotensin II-mediated cell proliferation

As Ang II enhances proliferation of VSMC^28^, we assessed the effect of Cu^(II)^ATSM pre-treatment on Ang II-mediated HCASMC proliferation (Fig. 5E). Cu^(II)^ATSM treatment (1 µM, 72 h) did not affect HCASMC number compared to control, providing further evidence that Cu^(II)^ATSM alone did not alter cell viability. Ang II treatment (200 nM, 72 h) increased proliferation 2.5 fold, which was significantly attenuated in cells pre-treated with Cu^(II)^ATSM (1 µM, 12 h).

### Cu^(II)^ATSM reduces angiotensin II-induced intracellular [Ca^2+^] increases

Ang II increases [Ca^2+^]_i_ in smooth muscle cells^27^, leading to increased superoxide generation^2^. We thus determined whether Cu^(II)^ATSM alters Ang II-mediated changes in intracellular [Ca^2+^] in HCASMC (Fig. 5F). A significant increase in [Ca^2+^]_i_ was observed in HCASMC treated with Ang II (200 nM, 30 min), however pre-treatment with Cu^(II)^ATSM (1 µM, 12 h) significantly attenuated Ang II-induced [Ca^2+^]_i_ increases without affecting basal [Ca^2+^]_i_. As DJ-1 is required for Nrf2 stability, and has also been reported to play a role in [Ca^2+^] handling^47^, we assessed Ang II-induced [Ca^2+^]_i_ following DJ-1 knockdown in HCASMC. DJ-1 knockdown abolished Cu^(II)^ATSM-mediated protection against Ang II-induced increases in intracellular [Ca^2+^]_i_.

### Cu^(II)^ATSM increases protein association of DJ-1 with SOD1 and intracellular Cu^(II)^ levels

**Figure 6:**
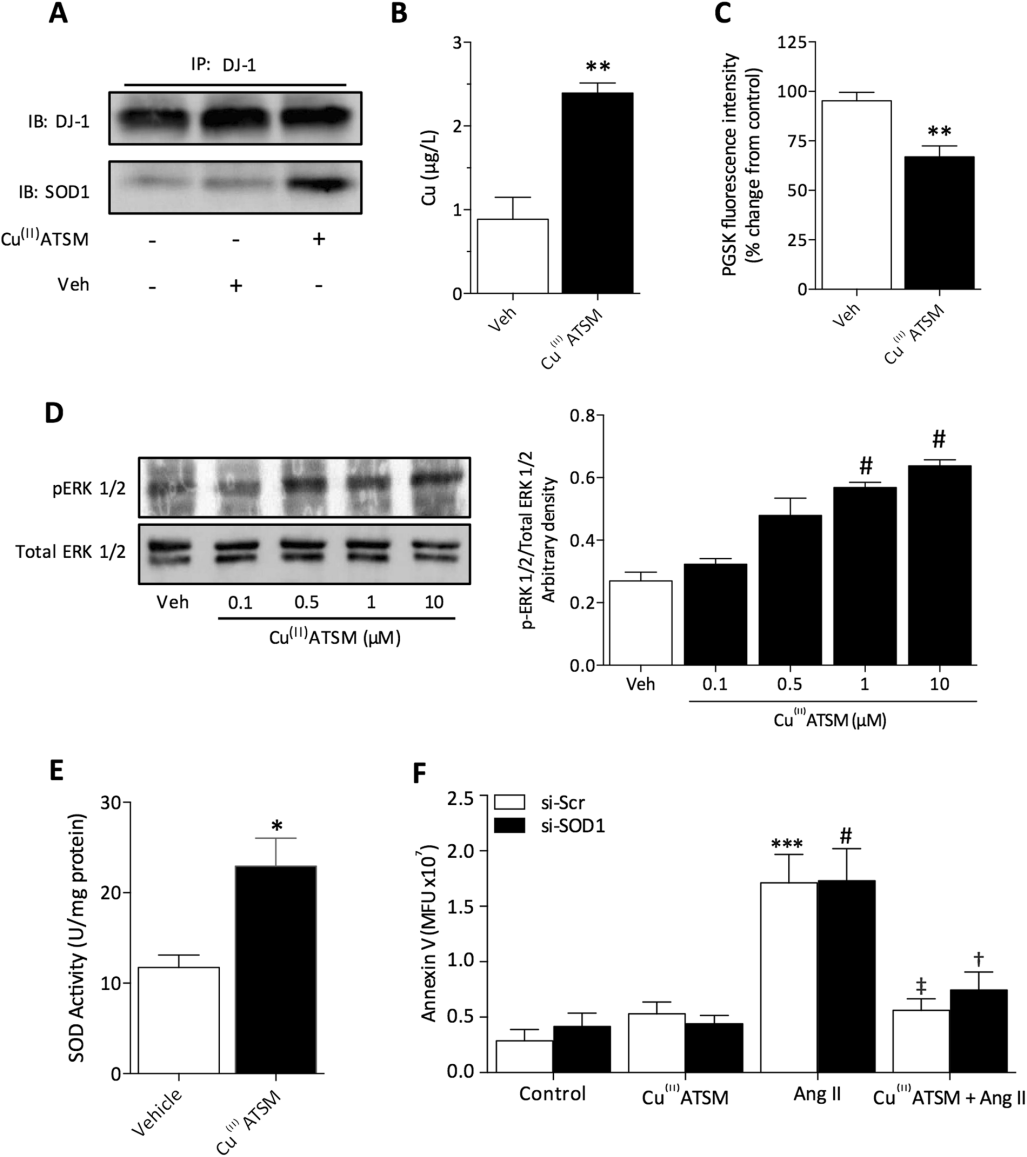
Cu^(II)^ATSM increases association of DJ-1 with SOD1, intracellular Cu^(II)^ levels and SOD activity in HCASMC. (**A**) HCASMC were treated with Cu^(II)^ATSM (1 µM, 1 h), immunoprecipitated for DJ-1 and immunobloted for DJ-1 and SOD1 (representative of n = 4 donors). Intracellular levels of Cu^(II)^ were assessed in HCAMSC treated with Cu^(II)^ATSM (1 µM, 30 min) using either ICP-MS (**B**) or Phen Green SK fluorescence (**C**). Data denote mean ± S.E.M., n = 4, **P < 0.01 vs Veh, (Students’ t-test). (**D**) HCASMC were treated with Cu^(II)^ATSM (0.1, 0.5, 1 and 10 µM, 15 min) and phosphorylation of ERK 1/2 detected by immunoblotting and analysed by densitometry relative to α-tubulin. Data denote mean ± S.E.M., n = 4, *P < 0.05, **P < 0.001 vs vehicle (one-way ANOVA and Bonferroni *post hoc* analysis). (**E**) HCASMC were treated with Cu^(II)^ATSM (1 µM, 30 min) and SOD1 activity assessed. Data denote mean ± S.E.M., n = 4, *P < 0.05 vs Veh, (Students’ t-test). (**F**) Annexin V fluorescence to assess Ang II (200 nM, 12 h) induced apoptosis in HCASMC following SOD1 knockdown by siRNA and pre-treatment with Cu^(II)^ATSM (1 µM, 12 h). Data denote mean ± S.E.M., n = 4, ***P < 0.001 vs si-Scr control, ^#^P < 0.001 vs si-SOD1 control, ^‡^P < 0.001 vs si-Scr cells treated with Ang II in the absence of Cu^(II)^ATSM, ^†^P < 0.001 vs si-SOD1 cells treated with Ang II in the absence of Cu^(II)^ATSM (one-way ANOVA and Bonferroni post hoc analysis).

DJ-1 has been demonstrated to act as a Cu^(II)^ chaperone, which has been directly associated with an increase in its association with SOD1 and its enzyme activity^11^. We therefore hypothesized that acute treatment with Cu^(II)^ATSM increases the association of DJ-1 with SOD1 in HCASMC. Immunoprecipitation experiments confirmed that treatment of HCASMC with Cu^(II)^ATSM (1 µM, 30 min) significantly increased the association of DJ-1 with SOD1 (Fig. 6A). Our data clearly demonstrate that DJ-1 is not only involved in the induction of Nrf2-regulated antioxidant enzymes, but can also enhance SOD1 association with DJ-1 following acute Cu^(II)^ATSM treatment. This suggests that Cu^(II)^ binding by DJ-1 may mediate both SOD1 and Nrf2 activation. Furthermore, the increased association between DJ-1 and SOD1 suggests that DJ-1 was enriched with Cu^(II)^ through an increase in intracellular Cu^(II)^ levels^11^. We determined whether Cu^(II)^ATSM increases intracellular Cu^(II)^ using both inductively coupled plasma-mass spectrometry (ICP-MS, Fig. 6B) and Phen Green SK (PGSK) fluorescence (Fig. 6C). A significant increase in intracellular Cu^(II)^ was observed following acute Cu^(II)^ATSM (1 µM, 30 min) treatment, suggesting that augmented Cu^(II)^ levels may mediate the effects of Cu^(II)^ATSM to increase SOD1 activity through DJ-1 association and antioxidant enzyme expression via DJ-1/Nrf2 signaling. We further report a significant increase in ERK1/2 phosphorylation (Fig. 6D), which has been implicated in the dissociation of Cu^(II)^ from ATSM^29^, suggesting bioavailable Cu^(II)^ is increased in HCASMC treated acutely with Cu^(II)^ATSM (1 µM, 15 min). In addition to the increased association between SOD1 and DJ-1, we also observed a 2-fold increase in SOD1 activity (Fig. 6E), providing further evidence that acute Cu^(II)^ATSM activates SOD1 activity, thereby acutely reducing superoxide generation. However, the acute protection afforded by Cu^(II)^ATSM does not affect the cytoprotection observed following Cu^(II)^ATSM pre-treatment, as protection against Ang II-induced apoptosis remains unaltered after SOD1 knockdown (Fig. 6F), suggesting that Cu^(II)^ATSM provides protection via two independent pathways.

## Discussion

Current therapeutic strategies have had limited success in augmenting endogenous antioxidant defenses to counteract oxidative stress in cardiovascular diseases. Although recent findings have established that Cu^(II)^ATSM affords protection against oxidative stress in the brain^13,14^, the underlying molecular mechanisms remain to be elucidated. Our study provides novel evidence that both oral delivery of Cu^(II)^ATSM in mice, and *in vitro* Cu^(II)^ATSM treatment of HCASMC and HCM, significantly upregulates Nrf2 dependent antioxidant defenses which is likely to confer protection against cardiovascular diseases associated oxidative stress^7^.

Classically, modification of Keap1 cysteine residues by oxidative or electrophilic stress inhibits proteasomal degradation of Nrf2^30^. The electrophilic nature of dietary compounds such as sulforaphane and curcumin makes them suitable Nrf2 activators^31^, however, it remains to be determined whether Cu^(II)^ATSM, a neutral and lipophilic compound^15^ (Fig. S1A) is able to activate Nrf2 via interactions with Keap1. Although Cu^(II)^ can mediate Nrf2 activation via a redox-cycling mechanism^32^, the levels of free Cu^(II)^ in our study are likely to be lower compared to previous reports using compounds that can release significantly higher levels of Cu^(II)^ under normal cell culture conditions compared to levels achieved by Cu^(II)^ATSM^33^. The intracellular dissociation of Cu^(II)^ATSM has been shown to increase the phosphorylation of ERK1/2 within a hypoxic environment^29^. We demonstrate that Cu^(II)^ATSM treatment enriches Cu^(II)^, in HCASMC and enhances ERK1/2 phosphorylation, suggesting an increase in bioavailable Cu^(II)^ ^29^. Moreover, ERK1/2 mediates Nrf2 phosphorylation at serine 40 and its activation^21^, providing an additional mechanism through which Cu^(II)^ATSM may enhance Nrf2 signaling.

Although the Parkinson’s associated protein DJ-1 is required for Nrf2 stability^10,34,35^, the presence of conserved cysteine residues on DJ-1 suggests a role as a redox sensor^36–39^, which may additionally modulate Nrf2 activity^24^. Copper chaperone functionality of DJ-1 may further serve as a mechanism to activate Nrf2 following Cu^(II)^ATSM delivery^10^. It is possible that DJ-1 enriched by copper enhances Nrf2 activation, as the induction of antioxidant enzymes was only evident upon treating cells with the Cu^(II)^ATSM complex and not with the ATSM ligand alone. Notably DJ-1 has been shown to directly regulate SOD1 activity^11^. Cu^(II)^ATSM delivery *in vivo* has been reported to increase mutated SOD1 activity in the brain^13^, but to date, only experiments without the use of cells have established that copper enriched DJ-1 directly increases SOD1 activity^11,40,41^. In this study, we have identified increased DJ-1 and SOD1 protein interactions in HCASMC treated with Cu^(II)^ATSM, providing a possible mechanism by which SOD1 activity may be increased acutely. Furthermore, we also demonstrate that acute Cu^(II)^ATSM-mediated protection via SOD1 occurs in addition to the activation of Nrf2 and target antioxidant defense proteins conferring long term protection.

Hearts of DJ-1 deficient mice have been shown to exhibit increased cardiomyocyte apoptosis, excessive DNA oxidation and cardiac hypertrophy when subjected to trans-aortic banding, as well as increased oxidative stress in response to Ang II infusion^19^, suggesting an important role for DJ-1 in cardioprotection. Notably, renal depletion of DJ-1 in mice decreases Nrf2 expression and activity, leading to increased oxidative stress and elevated systolic blood pressure^42^. Knockdown of renal Nrf2 in mice increases systolic blood pressure without effecting DJ-1 expression, suggesting that Nrf2 activation is downstream of DJ-1 and is thus required for the maintenance of redox balance. Our data corroborate these findings, as Cu^(II)^ATSM was unable to induce HO-1 and NQO1 expression in HCASMC following DJ-1 knockdown. Furthermore, our observation that Cu^(II)^ATSM increases DJ-1 expression suggests that this multifunctional protein is involved in the therapeutic protection by Cu^(II)^ATSM against cardiovascular oxidative stress, in part through its ability to stabilise Nrf2^10^ and enhancing the expression of endogenous antioxidant enzymes.

Recent studies have shown that Cu^(II)^ containing compounds have a therapeutic potential in inflammation, cancer, cardiac hypertrophy, PD and other neurodegenerative disorders^14,43–45^, suggesting that Cu^(II)^ATSM may additionally exhibit cardioprotective properties. Studies where Cu^(II)^ATSM has been orally administered in rodent models of ALS and PD have reported improved neurological outcomes and increased survival through the reduction of oxidative stress^13–15,44^. Although it has been reported that acute Cu^(II)^ATSM treatment reduces lipid peroxidation in an isolated perfused rat heart model of ischemia-reperfusion^46^, the underlying mechanisms were not determined. Therefore, our study provides novel mechanistic insights for the actions of Cu^(II)^ATSM to mediate cardiovascular protection via activation of Nrf2/DJ-1 signaling and induction of Nrf2-regulated antioxidant defenses.

Ang II contributes to the development and progression of hypertension and cardiovascular pathologies via increases in superoxide generation, intracellular [Ca^2+^] and cell proliferation^1,2,20^. Our findings in HCASMC strongly suggest that the observed protection against the pro-oxidant effects of Ang II on enhanced intracellular [Ca^2+^] and proliferation are conferred through the activation of Nrf2/DJ-1 signaling. The attenuation of Ang II-induced increases in [Ca^2+^]_i_, following pre-treatment of HCASMC with Cu^(II)^ATSM, is likely to decrease smooth muscle contractility associated with Ang II-mediated oxidative stress^1,2^. Notably, DJ-1 deficient mice exhibit altered Ca^2+^ homeostasis in skeletal muscle^47^, suggesting an additional role for DJ-1 in the redox regulation of [Ca^2+^]_i_ in HCASMC.

Smooth muscle apoptosis has been implicated in a number of processes contributing to cardiovascular diseases, including atherosclerotic plaque instability and rupture leading to myocardial infarction or cerebral stroke^48–50^. Ang II induces SMC apoptosis via activation of the Ang II type 2 receptor^27^, leading to enhanced caspase 3 activity, increased DNA fragmentation and oxidative stress^27,49,51^. We demonstrate that Cu^(II)^ATSM pre-treatment significantly attenuates Ang II-induced apoptosis in HCASMC, which was abolished following Nrf2 knockdown, suggesting that Nrf2-mediated upregulation of antioxidant enzymes may account for the protection afforded by Cu^(II)^ATSM. As DJ-1 knockdown also attenuated the protection afforded by Cu^(II)^ATSM against Ang II-induced apoptosis, it is likely that Nrf2-mediated antioxidant gene induction is also dependent on DJ-1 expression.

Oral delivery of Cu^(II)^ATSM in a mouse model of ALS markedly reduces levels of oxidatively modified protein carbonyls^15^. Cu^(II)^ATSM treatment in a mouse model of PD has also been linked to a significant reduction in oxidative stress, and thereby preventing aggregation of α-synuclein^14^. The similarities in the involvement of oxidative stress in both neurological and cardiovascular diseases highlights the therapeutic potential of Cu^(II)^ATSM in these pathologies. Although the protective properties of Cu^(II)^ATSM have been reported in rodent models of neurodegeneration, we now provide the first evidence that Cu^(II)^ATSM enhances cardiac and aortic expression of antioxidant proteins *in vivo* and provides protection against Ang II-mediated oxidative stress in HCASMC via Nrf2-regulated antioxidant defenses (Fig. 6).

Our study further confirm the potential therapeutic properties of Cu-containing compounds^52^ and is the first to demonstrate that Cu^(II)^ATSM induces antioxidant enzymes *in vivo* and in HCASMC and HCM *in vitro* via Nrf2/DJ-1 axis to protect against Ang II-mediated oxidative stress. Therefore, Cu^(II)^ATSM represents a novel Nrf2 and DJ-1 activator with therapeutic potential to enhance endogenous antioxidant defenses, providing protection against cardiovascular diseases through ameliorating oxidative stress.

## Material and Methods

### Treatment of animals

Male C57BL6 mice (6–8 weeks, Charles River, UK) were acclimatized for at least 1 week before treatment and maintained on a 12 h light/dark cycle. All procedures were approved by the UK Home Office and King’s College London after a rigorous ethical review process and performed under the authority of Project Licence No. PPL70/6579, in accordance with the UK Animal (Scientific Procedures) Act 1986. A suspension of the compound was prepared in standard suspension vehicle [SSV; 0.9% (w/v) NaCl, 0.5% (w/v) Na-carboxymethylcellulose (medium viscosity), 0.5% (v/v) benzyl alcohol, and 0.4% (v/v) Tween-80]. Cu^(II)^ATSM in SSV was delivered by oral gavage at a dose of 30 mg/kg body weight and the heart and aorta were harvested after 24 h. Control mice received an equivalent volume of SSV only.

### Culture of primary human coronary artery smooth muscle cells (HCAMSC) and cardiomyocytes (HCM)

Primary HCASMC from 4 male donors, and HCM from 2 male donors were obtained from PromoCell (Germany) or Lonza (USA). Cells were cultured in phenol red free basal medium (PromoCell, Germany) supplemented with fetal calf serum (5%), epidermal growth factor (0.5 ng/mL), basic fibroblast growth factor (2 ng/mL) and insulin (5 µg/mL). Confluent cultures at passage 4–8 were equilibrated in phenol red free basal medium supplemented only with 5% FCS (Sigma, UK), without growth factors for 24 h prior to treatments with Cu^(II)^ATSM (0.1 µM–10 µM), synthesised as previously described^53^. Replicate experiments were performed on cells from different donors where possible.

### Measurement of intracellular glutathione, ATP and cell viability

Intracellular GSH and ATP were extracted using 6.5% trichloroacetic acid (TCA, Sigma, UK). Intracellular GSH levels were assessed using a fluorometric assay as previously described^54^. For ATP measurement, extracts were incubated with firefly lantern extract (Sigma, UK) containing both luciferase and luciferin. GSH fluorescence and ATP luminescence were measured using a microplate reader (BMG Labtech ClarioStar, Germany). Cell viability was determined by assessing mitochondrial dehydrogenase activity using 3-[4,5-dimethylthiazol-2-yl]2,5-diphenyl tetrazolium bromide (MTT, Sigma, UK).

### Immunoblotting

Cells were lysed in SDS buffer and protein content was determined using the bicinchoninic acid assay. Proteins were separated by SDS-PAGE and membranes probed with HO-1 (BD Biosciences, UK), GCLM (gift of T. Kavanagh, University of Washington, WA, USA), Nrf2 (Santa Cruz, USA), phosphorylated (Ser40) Nrf2 (Abcam, UK), SOD1 (Abcam, UK), DJ-1 (Cell Signaling, USA), phosphorylated extracellular regulated kinase-1/2 (ERK1/2, Promega, UK), Total ERK 1/2 (Millipore, UK) and α-tubulin (Millipore, UK) antibodies. Horseradish-peroxidase-conjugated secondary antibodies were used with enhanced chemiluminescence (Millipore, UK) to visualise bands which were quantified by densitometry (Image J, NIH, USA).

### Assessment of Proliferation

For proliferation studies, HCASMC were seeded at 10,000 cells/well and cell number determined after 72 h treatment using a Neubauer haemocytometer.

### si-RNA mediated knockdown of Nrf2, DJ-1 and SOD1

Cells were seeded at 35,000 cells/well and transfected with 40 pmol/24 well of either scrambled si-RNA, Nrf2 si-RNA^55^, DJ-1 si-RNA or SOD1 si-RNA (Santa Cruz, USA) for 24 h with Dharmafect 1 transfection reagent (GE Healthcare, USA).

### Nrf2 immunofluorescence

HCASMC were treated with Cu^(II)^ATSM (1 µM, 1–4 h), fixed with paraformaldehyde (4%), permeabilized with Triton X-100 (0.1%) and Nrf2 immunofluorescence determined using a rabbit anti-Nrf2 antibody (Santa Cruz, USA) and Alexa Fluor 488 conjugated antibody (Life Technologies). Nuclei were labelled using Hoechst 33342 (Sigma, UK). Cells were visualised using a Nikon Diaphot microscope adapted for fluorescence (Nikon, Japan) and images acquired using a cooled CCD camera (Hamamatsu, Japan).

### Co-immunoprecipitation of DJ-1 and SOD

Cells were lysed with RIPA buffer (Sigma, UK). A rabbit DJ-1 antibody (Cell Signaling, USA) was incubated with Protein A beads (Biorad, UK) and complexes washed with PBS-0.1% Tween20. The antibody-bead complex was incubated with 100 µg cell lysates (1 h, 20 °C) and washed with PBS-0.1% Tween20. Immunoprecipitates were eluted following incubation of samples with 1x Laemmli buffer (70 °C, 10 min).

### Measurement of intracellular Cu^(II)^

Cu^(II)^ content of cells was determined by inductively coupled plasma-mass spectrometry (ICP-MS) as previously described^29^. After treatment of cells with Cu^(II)^ATSM (1 μM, 1 h), cells were lysed in 65% HNO_3_ (Sigma). Cu^(II)^ content was determined by ICP-MS (Perkin Elmer NexION 350D). Intracellular Cu^(II)^ levels were also assessed using Phen Green SK (PGSK, Life Technologies) as previously described^56^. Cells were loaded with PGSK (20 µM) for 30 min at 37°C, and incubated with Cu^(II)^ATSM (1 µM, 30 min). Fluorescence intensity (Ex:Em 490:510 nm) was determined using a microplate reader (BMG Labtech Clariostar, Germany).

### Measurement of intracellular [Ca^2+^]

HCASMC were loaded with Fura 2-AM (2 µM, Teflabs, USA) for 30 min at 37 °C in medium. Cells were then incubated in Krebs buffer and Fura-2AM fluorescence (excitation 340 and 380 nm, emission 520 nm) measured using a fluorescent plate reader (BMG Labtech Clariostar, Germany). Intracellular [Ca^2+^] was calculated using the Grynkiewicz formula^57^.

### Superoxide dismutase activity and superoxide generation

SOD1 activity was assessed using a commercially available SOD activity assay kit (Cayman Chemicals, USA). Total cellular superoxide production was assessed by L-012 enhanced chemiluminescence in live HCASMC cultures, as previously described^58^. Cells were incubated at 37 °C in Krebs buffer containing L-012 (20 µM). Luminescence was monitored over 10 min after 30 min equilibration at 37 °C in a luminescence microplate reader (Chameleon V, Hidex, Finland).

### Detection of mitochondrial superoxide generation

Mitochondrial superoxide production was measured using MitoSOX Red (Life Technologies, USA) as previously described^59^. Cells were loaded with MitoSOX Red (5 µM, 30 min) at 37 °C, fixed with 4% paraformaldehyde and visualised by fluorescence microscopy. Equivalent numbers of cells were imaged in each field. Fluorescence intensity per cell was corrected for background intensity and quantified using image analysis software (Image J, NIH, USA)^55,59^.

### Assessment of apoptosis

Annexin V binding to phosphatidylserine can be used as a marker of early apoptotic events^60^. Binding of Cy5-conjugated annexin V to HCASMC was assessed using a kit (Biotium, USA). Cells were co-stained with Hoechst 33342 (Sigma, UK) to identify nuclei and visualised using a fluorescence microscope (Nikon, Japan) and images acquired using a cooled CCD camera (Hamamatsu, Japan). Equivalent numbers of cells were captured for each field. Fluorescence intensity was determined using analysis software (Image J, NIH, USA).

### Statistical analysis

Data denote mean ± S.E.M. of experiments. All experiments were performed in n = 4–8 different cultures of HCASMC (from 4 donors) or HCM (from 2 donors). Comparison of more than two conditions in the same experiment were evaluated using either a Student’s t-test or one way or two-way ANOVA followed by Bonferroni *post hoc* test. P < 0.05 values were considered significant.

## Electronic supplementary material

Supplementary Information
