# Supplementary material for: Cardioprotective effects of Cu(II)ATSM in human vascular smooth muscle cells and cardiomyocytes mediated by Nrf2 and DJ-1
Source: Sci Rep. 2016 Dec 21;6:7. doi: 10.1038/s41598-016-0012-5 (PMC5431352; doi:10.1038/s41598-016-0012-5)
Supplement: Supplementary file 1 — Supplementary Information [file 41598_2016_12_MOESM1_ESM.pdf]

Cardioprotective effects of $Cu^{(II)}$ATSM in human vascular smooth muscle cells and cardiomyocytes mediated by Nrf2 and DJ-1

Salil Srivastava[1], Philip J. Blower[2], Aisah A. Aubdool[1], Robert C. Hider,[3]

Giovanni E. Mann[1§], Richard C. Siow[1§]*

[1]Cardiovascular Division and [2]Imaging Sciences & Biomedical Engineering Division, British Heart Foundation Centre of Research Excellence, [3]Institute of Pharmaceutical Science, Faculty of Life Sciences & Medicine, King's College London, 150 Stamford Street, London SE1 9NH, U.K.

Supplementary Material

[§]Joint Senior Authors

Running Title: Cardiovascular protection by $Cu^{(II)}$ATSM via Nrf2/DJ-1

***Correspondence to**:

Dr Richard Siow, Cardiovascular Division, BHF Centre of Research Excellence, Faculty of Life Sciences & Medicine, King's College London, Franklin-Wilkins Building, 150 Stamford Street, London SE1 9NH, U.K.

Tel. +44 (0)20 7848 4333; Fax +44 (0)20 7848 4500
Email: richard.siow@kcl.ac.uk

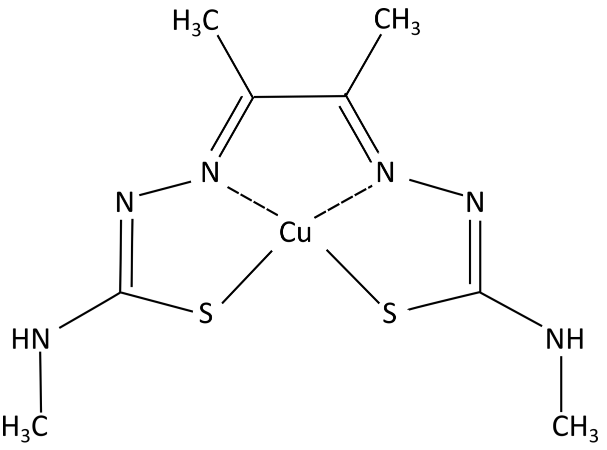

Figure S1. Chemical structure of Cu$^{(II)}$ATSM

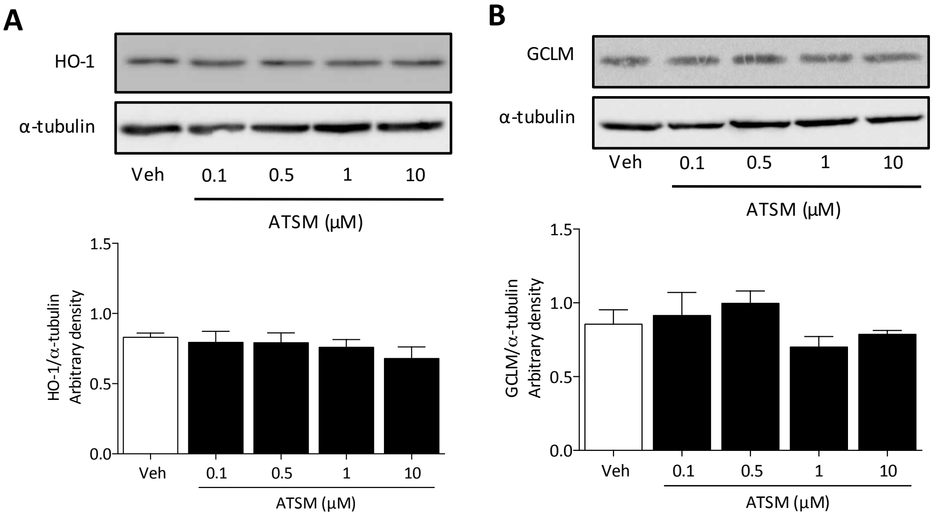

Figure S2. ATSM does not induce Nrf2-regulated HO-1 and GCLC expression. HCASMC were treated with the bis(thiosemicarbazone) ligand ATSM (0.1, 0.5, 1 and 10 μM, 12h). Expression of (**A**) HO-1 and (**B**) GCLM were determined by immunoblotting relative to α-tubulin. Data denote mean ± S.E.M, n=4 (one-way ANOVA and Bonferroni *post hoc* analysis).

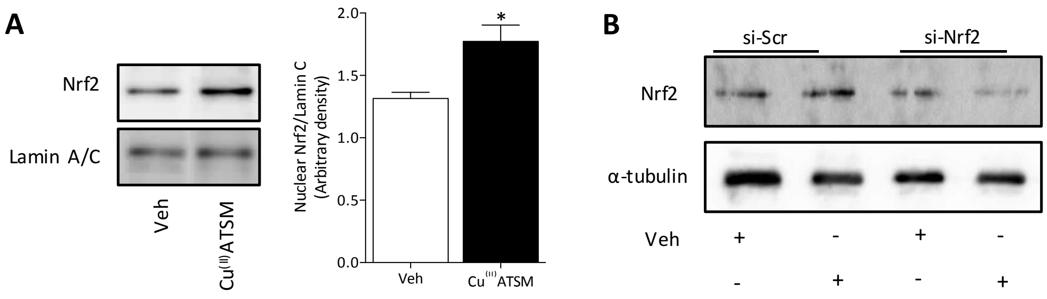

Figure S3. Cu$^{(II)}$ATSM induces nuclear translocation of Nrf2 in HCASMC and confirmation of Nrf2 knockdown by siRNA. (**A**) Nuclear translocation of Nrf2 was assessed using nuclear lysates from HCASMC treated with Cu$^{(II)}$ATSM (1μM, 4h) determined by immunoblotting relative to Lamin C. Data denote mean ± S.E.M, n=4, *P<0.05 (Students' t-test). (**B**) Representative immunoblot confirming Nrf2 knockdown in HCASMC following transient transfection using siRNA.

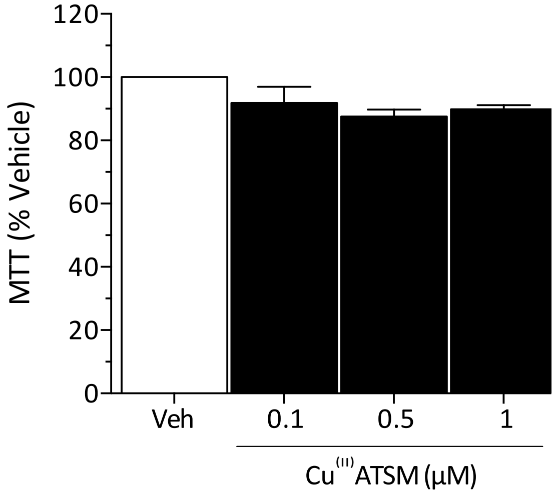

Figure S4. $Cu^{(II)}$ATSM does not affect cell viability. HCASMC were treated with Cu(II)ATSM (0.1 – 1μM, 24h) and cell viability assessed using MTT. Data expressed as percentage change from vehicle and denote mean ± S.E.M, n=4 (one-way ANOVA and Bonferroni *post hoc* analysis).

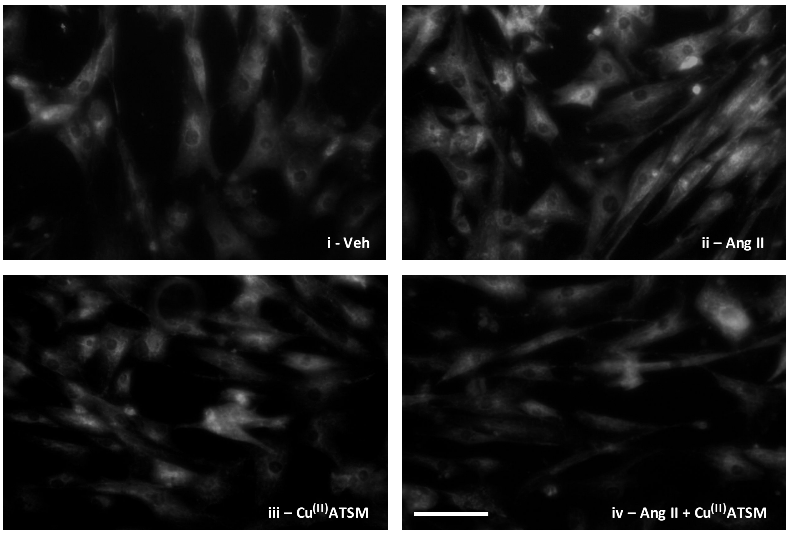

Figure S5. Cu$^{(II)}$ATSM pre-treatment attenuates Ang II-induced mitochondrial superoxide generation. HCASMC were pre-treated with Cu$^{(II)}$ATSM (1µM, 12h) prior to treatment with angiotensin II (Ang II, 200 nM, 4h) and MitoSOX red fluorescence imaged. A minimum of 5 fields of view were captured per condition from 4 different cultures. Equivalent cell numbers were captured in each field of view. Scale bar represents 5 µm.

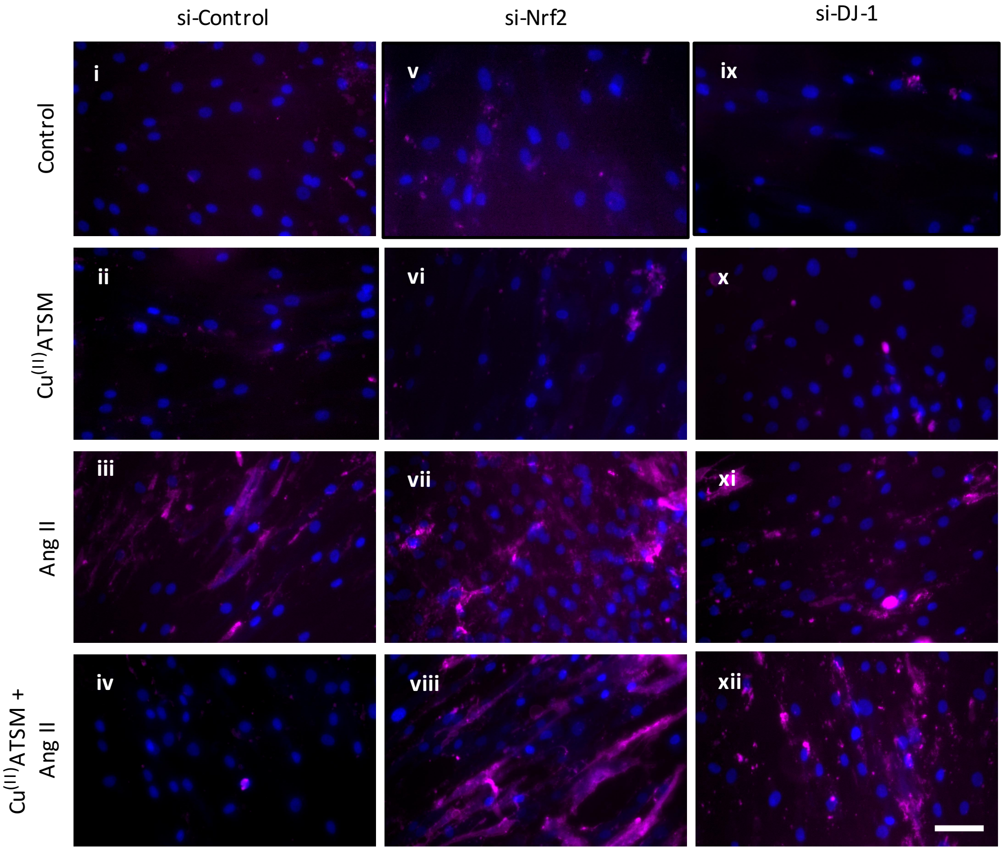

Figure S6. Cu$^{(II)}$ATSM pre-treatment protects against Ang II-induced apoptosis. Annexin V fluorescence was measured to assess Ang II (200 nM, 12h) induced apoptosis in HCASMC following Nrf2 or DJ-1 siRNA knockdown, then pre-treated with vehicle or Cu$^{(II)}$ATSM (1µM, 12h) and challenged with Ang II. Apoptotic cells exhibit annexin V (purple) staining and Hoechst 33342 was used to identify nuclei (blue). A minimum of 5 fields of view were captured per condition from 4 different cultures. Equivalent cell numbers were captured in each field of view. Scale bar represents 5µm.
